# Myc on my mind: a transcription factor family's essential role in brain development

**DOI:** 10.18632/oncotarget.113

**Published:** 2010-06-13

**Authors:** Lori A. Mainwaring, Bobby Bhatia, Anna Marie Kenney

**Affiliations:** Department of Cancer Biology and Genetics, Memorial Sloan-Kettering Cancer Center, New York, NY 10021 USA

Researchers have been captivated by the myc family of proto-oncogenes and their ability to regulate a diverse array of cellular processes through transcriptional regulation of target genes. Since the early 1980s researchers have worked to understand how Myc family members regulate hundreds of genes in a context-dependent and cell type-specific manner. Gene expression profiling performed in various over-expression platforms have identified hundreds of genes regulated by Myc but have done little to narrow the focus of Myc's function as the genes typically fall into multiple biological categories, including protein biosynthesis, metabolism, cell cycle regulation and microRNAs [1, 2]. In order to parse the cell type-specific function of Myc proteins, future studies will benefit from the generation of mouse models wherein gene manipulation can be restricted to specific cell types.

Mammals possess three closely related myc family members: c-, N-, and L-myc, which have developmentally regulated, tissue specific expression patterns. These proteins, basic-helix-loop-helix zipper (bHLHZ) transcription factors, heterodimerize with the ubiquitously-expressed Max protein to activate gene expression. In complex with another protein, Miz1, myc may suppress gene expression [3, 4]. Myc has been predicted with gene promoters, including those that control cell cycle regulation, apoptosis, differentiation programs as well as chromatin remodeling [1, 5]. More recently Myc proteins have also been shown to regulate genes involved in ribosome biosynthesis and other components involved in protein translation [6], suggesting that Myc's roles in cellular processes extend beyond cell cycle and apoptosis regulation. Furthermore, Myc's ability to cooperate with three other transcription factors (Oct4, Sox2 and Klf4) to reprogram somatic cells to induced pluripotent stem cells suggests Myc proteins have critical roles in maintaining cells in an undifferentiated state [7, 8].

**Fig.**
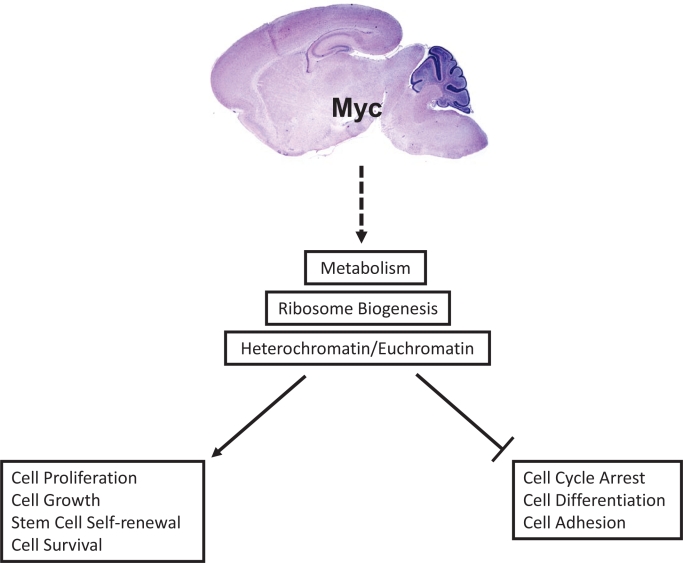
Myc controls multiple signaling pathways Myc proteins increase euchromatin levels, which is associated with gene transcription and the induction of metabolism, ribosome biogenesis, and protein synthesis, resulting in cellular proliferation, growth, survival, and stem cell self -renewal. Loss or suppression of Myc changes heterochromatin levels, which leads to the suppression of transcription and ribosome biogenesis and protein synthesis, resulting in cell-cycle arrest, differentiation, and cell adhesion.

The role for Myc proteins in proliferation has been well documented in mammals through embryonic and conditional knockout mouse models. c-myc is expressed widely in the mouse embryo and expression patterns correlate to rapidly proliferating cells [9]. Accordingly, constitutive c-myc knockout mice do not survive past embryonic day (E) 10.5 due to an overall reduced size, enlarged hearts, and a failure in neural tube closure [10]. N-myc expression on the other hand, is restricted primarily to epithelial tissues especially those of the developing nervous system. Homozygous N-myc mutant embryos die around E11.5 as a result of defects in the central and peripheral nervous system as well as poorly developed lung and gut tissue [11]. Because of embryonic lethality of c- and N-myc null mice, tissue specific deletions of Myc genes have been generated and proved to be usefully in demonstrating the importance of these genes in proliferation, which is illustrated by c-myc loss in hematopoietic cells [12, 13].

The role of N-myc in neural progenitor cells has been characterized by targeted deletion of the gene in Nestin-expressing cells. In addition to overall brain growth defects, the authors observed profound disruptions in cerebellar development as a consequence of reduced numbers of neural progenitors cells and decreased proliferation rates in cerebellar precursor cells [14]. Interestingly knockout of c-myc in neural progenitors resulted in only moderate growth defects [14, 15]. Taken together these single myc gene conditional knockout models suggest that c-myc and N-myc have compensatory roles but also have individual functions that are important in the developing nervous system.

In order to dissect out how c-myc and N-myc cooperate to promote neural development, Wey and Knoepfler deleted both genes in Nestin-expressing neural precursor cells. The double knock-out (DKO) mice had a more severe phenotype compared to the single Myc gene conditional knockouts and the growth defects were predominately observed in the forebrain/neocortex and hindbrain. The growth defects were determined to be a result of decreased mitotically active cells as well as a failure of progenitor cells in the subventricular and ventricular zone to initiate differentiation programs. Surprisingly, gene expression profiling from the DKO mouse brains revealed modest levels of changes in gene expression compared to controls; however, specific patterns of changes were observed based on ontological analysis of the genes that were significantly altered. A cluster of mitosis-related genes was significantly down-regulated in the DKO mice further indicating a critical role for myc genes in neural progenitor proliferation. Consistent with previous studies, genes involved in cellular metabolism and ribosome biogenesis were also affected in DKO mice [16, 17]. The characterization and analysis of the DKO mice confirm the importance of Myc activity in neural precursor proliferation and identify potential Myc targets that may function to regulate multiple cellular processes. However, further studies will be required to validate the candidate regulated genes as bona fide myc targets, as well as additional mechanistic analysis to verify the roles of myc in the biological processes proposed to comprise the myc transcriptional program during brain development.

The report by Wey and Knoepfler sheds light into the role of myc genes in the developing nervous system but also reveals a useful, important genetic tool for studying myc gene targets in the context of specific brain regions or cellular processes. The mice harboring conditionally ablatable c- and/or N-myc will be amenable to in vitro studies of primary neural precursors, neural stem cells, and brain slice explants which can then be exposed to Cre recombinase-carrying vectors, for example, to provide insight into the mechanisms by which myc regulates neural progenitor proliferation and differentiation. Furthermore the DKO mice will be useful in conjunction with the single myc gene conditional KO mice to look at differences in gene expression profiles and for identification of gene sets critical for proliferation, cell growth, or differentiation in the context of tumors arising from neural stem/progenitor cell populations.
